# Influence of winding number on vortex knots dynamics

**DOI:** 10.1038/s41598-019-53548-w

**Published:** 2019-11-21

**Authors:** Chiara Oberti, Renzo L. Ricca

**Affiliations:** 1 Department of Mathematics & Applications, University of Milano-Bicocca, Via Cozzi 55, 20125 Milano, Italy; 2 BDIC, Beijing University of Technology, 100 Pingleyuan, Beijing, 100124 P.R. China

**Keywords:** Fluid dynamics, Applied mathematics

## Abstract

In this paper we determine the effects of winding number on the dynamics of vortex torus knots and unknots in the context of classical, ideal fluid mechanics. We prove that the winding number — a topological invariant of torus knots — has a primary effect on vortex motion. This is done by applying the Moore-Saffman desingularization technique to the full Biot-Savart induction law, determining the influence of winding number on the 3 components of the induced velocity. Results have been obtained for 56 knots and unknots up to 51 crossings. In agreement with previous numerical results we prove that in general the propagation speed increases with the number of toroidal coils, but we notice that the number of poloidal coils may greatly modify the motion. Indeed we prove that for increasing aspect ratio and number of poloidal coils vortex motion can be even reversed, in agreement with previous numerical observations. These results demonstrate the importance of three-dimensional features in vortex dynamics and find useful applications to understand helicity and energy transfers across scales in vortical flows.

## Introduction

Work on vortex knots dates back to the earlier studies of Lord Kelvin and his visionary vortex atom theory^1^. After a long period of neglect the more recent discovery of knotted orbits in dynamical systems^2^ has stimulated new interest in the study of knotted fields^3–7^, particularly as regards classical and quantum vortex dynamics^8–15^. Work on determining the influence of geometric and topological features on the dynamics of vortex knots has been limited to approximated models, based either on the use of the so-called localized induction approximation (LIA)^16–19^ or regularized Biot-Savart law by cut-off methods^8,10,19,20^.

In this paper we provide a mathematical proof that the effects of winding number — a topological invariant of torus knots — are of primary importance on the motion of vortex knots in the context of classical, ideal (i.e. inviscid) fluid mechanics. This is done by considering thin-core vortex filaments in the shape of torus knots under the full Biot-Savart law. The influence of winding number is found to be comparable to curvature effects, and for thin filaments it is more important than the vorticity distribution over the vortex cross-section. We also determine for the first time, and in full generality, precise relations between winding number, knot complexity and relative velocity contributions. For most common knot types we compute the speed and show, as expected, that the propagation velocity increases with the number of toroidal coils. Moreover, we prove that for increasing aspect ratio and number of poloidal coils vortex motion can be even reversed.

Estimates of the self-induced velocity are notoriously difficult to be evaluated analytically because of the divergent behavior of the Biot-Savart integral when the induction point is asymptotically close to the vortex. In order to make analytical progress we assume uniform vorticity on a small, circular vortex cross-section, and vortex geometry given by parametric equations of knots standardly embedded on a mathematical torus. This allows us to take advantage of the rotational symmetry of these knot types, by reducing the Biot-Savart integral to a line integral, function solely of torus aspect ratio *λ* and winding number *w* (see the following section for definitions). The integral is then analytically de-singularized by applying the Moore-Saffman technique^21^ following the same procedure as in^22^. The functional dependence of finite terms on *λ* and *w*, and ultimately the influence of *λ* and *w* on the speed of the vortex, is thus determined. The new results are then compared with earlier results obtained numerically under various assumptions.

The paper is organized as follows. In the following first subsection  we introduce basic definitions and the parametric equations for torus knots, and in the second subsection the reduction of the Biot-Savart law to a line-integral. Then  we apply the de-singularization technique of Moore-Saffman to extract the finite term contribution in terms of *λ* and *w*. Next  we determine the influence of *λ* and *w* by analyzing 56 knots and unknots up to 51 crossings, determining the velocity components along tangent, normal and binormal directions. From these components we reconstruct the speed of the vortex, provide a criterium for the reversal of vortex motion and compare the results with previous works. Conclusions are drawn in the last section.

## Reduction of the Biot-Savart Induction Law for Torus Knots and Unknots

### Torus knots and unknots

**Figure 1:**
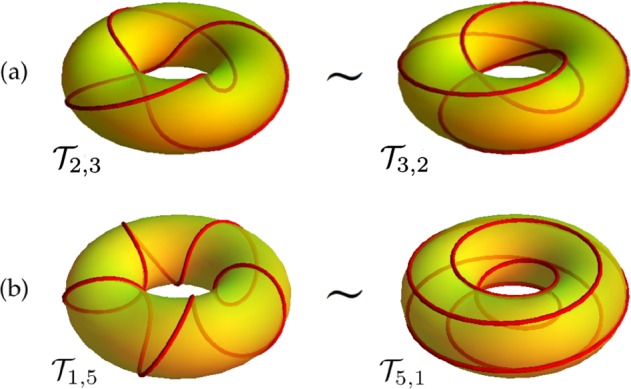
Torus knots and unknots (red online) on the mathematical torus *Π* (yellow). (**a**) Trefoil knot ${{\mathscr{T}}}_{\mathrm{2,3}}$ (left) and ${{\mathscr{T}}}_{\mathrm{3,2}}$; these two knots are topologically equivalent to each other, that is one can be deformed to the other by a series of continuous deformations. (**b**) Poloidal coil ${{\mathscr{T}}}_{\mathrm{1,5}}$ (left) and toroidal coil ${{\mathscr{T}}}_{\mathrm{5,1}}$; these are unknots topologically equivalent to the standard circle.

Torus knots and unknots are rotationally symmetric closed curves standardly embedded on a mathematical torus *Π* in ${{\mathbb{R}}}^{3}$ (see^23^). Each torus knot is defined by a pair of co-prime integers *p* > 1 and *q* > 1, denoting the number of full turns around *Π* done by the curve along the longitudinal (or toroidal) direction and meridian (or poloidal) direction, respectively (see Fig. 1a). The *unknot* is given by either *p* = 1 or *q* = 1 (see Fig. 1b). When *p* = *q* = 1 the unknot is a twisted circle on *Π*, and when *p* and *q* are rational, but not relatively prime, we have links (with number of components given by the greatest common divisor between *p* and *q*). The ratio *w* = *q*/*p* (*w* > 0) defines the *winding number* and is a measure of the knot topology. When *w* = *q*/*p* is irrational the curve forms a dense set covering *Π* almost everywhere. Two limits are of interest: (i) a *poloidal* hollow ring covered by infinitely many poloidal coils, when *p* is fixed and finite, and *q* → ∞; (ii) a *toroidal* hollow ring, when *q* is fixed and finite, and *p* → ∞.

There are several, equivalent, geometric parametrizations of torus knots. Since the longitudinal angle *α* and meridian angle *β* are related by *β* = *wα*, the vector position of points on ${{\mathscr{T}}}_{p,q}$ can be written as

1 $${{\bf{x}}}^{\ast }=R\mathrm{[(1}+\lambda \,\cos \,w\alpha )\,\cos \,\alpha ,\,\mathrm{(1}+\lambda \,\cos \,w\alpha )\,\sin \,\alpha ,\lambda \,\sin \,w\alpha ]$$

where *α* ∈ [0, 2*πp*), *R* and *r* the large and small radius of *Π* respectively (0 < *r* < *R*), and *λ* = *r*/*R* the torus *aspect ratio*, with *λ* ∈ (0, 1). The knot handedness is then prescribed by Eq. (1).

### Biot-Savart law for vortex knots: asymptotic formula and leading order terms

A vortex torus knot is a vortex filament of negligible cross-section identified with the knot type ${{\mathscr{T}}}_{p,q}$. We take the vortex to be embedded in an inviscid, irrotational, incompressible and unbouded fluid at rest at infinity, with vorticity ***ω*** = ∇ × **u** (**u** velocity) localized on a thin tube centred on ${{\mathscr{T}}}_{p,q}$. The Lagrangian *L* of the system is defined by  *L* = *E* − *U*, where *E* is the total energy (kinetic energy *K* plus potential energy *U*), so that for an isolated vortex in an unbouded fluid it simply coincides with the kinetic energy of the vortex, given by

2 $$K=\frac{1}{2}\int |{\bf{u}}({\bf{x}}{)|}^{2}{\rm{dV}},$$

where *V* is fluid volume. In absence of other contributions, the velocity **u** is solely that induced by vorticity through the inverse of the curl operator, given by the classical Biot-Savart law^24^. Note that since $|{\bf{u}}{|}^{2} \sim O(|{\bf{x}}{|}^{-6})$ as |**x**| → ∞, the integral (2) converges. Here we want to determine the self-induced motion of the vortex filament in steady conditions. For this we take vorticity ***ω*** = *ϖ*_0_$\hat{{\bf{t}}}$, with *ϖ*_0_ = constant and $\hat{{\bf{t}}}$ unit tangent to ${{\mathscr{T}}}_{p,q}$, with Frenet frame given by unit tangent, normal and binormal {$\hat{{\bf{t}}},\,\hat{{\bf{n}}},\,\hat{{\bf{b}}}$} on ${{\mathscr{T}}}_{p,q}$. For the assumptions made above the velocity **u** = **u**(**x**) induced by ***ω*** = ***ω***(${{\bf{x}}}^{\ast }$) (${{\bf{x}}}^{\ast }\in {{\mathscr{T}}}_{p,q}$) at any irrotational point ${\bf{x}}\in {{\mathbb{R}}}^{3}$ exterior to the vortex can thus be written in terms of a line integral, given by

3 $${\bf{u}}({\bf{x}})=\frac{\varGamma }{{\rm{4}}\pi }{\oint }_{{{\mathscr{T}}}_{p,q}}\frac{\hat{{\bf{t}}}({{\bf{x}}}^{\ast })\times ({\bf{x}}-{{\bf{x}}}^{\ast })}{|{\bf{x}}-{{\bf{x}}}^{\ast }{|}^{{\rm{3}}}}{\rm{d}}{{\bf{x}}}^{\ast },$$

where *Γ* is vortex circulation. The Hamiltonian (per unit density and appropriately regularised) associated with Eq. (3) is given by^25,26^

4 $$H=\frac{{\varGamma }^{2}}{4\pi }\mathop{\oint \,\,\oint }\limits_{{{\mathscr{T}}}_{p,q}}\,\frac{{\rm{d}}{{\bf{x}}}_{1}^{\ast }\cdot {\rm{d}}{{\bf{x}}}_{2}^{\ast }}{|{{\bf{x}}}_{1}^{\ast }-{{\bf{x}}}_{2}^{\ast }|}\,\mathrm{.}$$

Using (1), Eq. (3) becomes

5 $${\bf{u}}({\bf{x}})=\frac{\varGamma }{4\pi }{\int }_{0}^{2\pi p}\frac{\hat{{\bf{t}}}(\alpha )\times ({\bf{x}}-{{\bf{x}}}^{\ast }(\alpha ))}{|{\bf{x}}-{{\bf{x}}}^{\ast }(\alpha {)|}^{3}}|\frac{{\rm{d}}{{\bf{x}}}^{\ast }(\alpha )}{{\rm{d}}\alpha }|\,{\rm{d}}\alpha ,$$

where integration on *α* is extended to the number *p* of toroidal coils.

The propagation velocity of the vortex is given by considering the self-induction at a point **x**_*o*_ asymptotically close to the nearest source point ${{\bf{x}}}^{\ast }$, i.e. when **x** = **x**_*o*_ → ${{\bf{x}}}^{\ast }$. The asymptotic behavior of (3) was originally derived by Da Rios in 1911, re-formulated by Levi-Civita in 1932 (for a historical reconstruction see^27^) and subsequently re-discovered by Batchelor^24^. In general, for any point asymptotically close to the vortex the self-induced velocity is given by

6 $${\bf{u}}({{\bf{x}}}_{o})=\frac{\varGamma }{2\pi \sigma }\,\hat{{\bf{q}}}+\frac{\varGamma }{4\pi \rho }\,[{\rm{l}}{\rm{n}}\frac{\rho }{\sigma }+F]\,\hat{{\bf{b}}}+{\bf{G}}\,({{\bf{x}}}_{o}\to {{\bf{x}}}^{\ast }),$$

where *ρ* is the local radius of curvature, *σ* the radius of the vortex circular cross-section (with $\sigma \ll \rho$), $\hat{{\bf{q}}}$ the azimuthal unit vector, *F* some function of the local vorticity distribution, and **G** a finite term contribution that depends on far-field effects. Since the azimuthal term does not contribute to the displacement of the vortex, the drift velocity is given by **v**(**x**_*o*_) = **u**(**x**_*o*_) − [*Γ*/(2*πσ*)]$\hat{{\bf{q}}}$. It is well-known that when **x** = **x**_*o*_ → x^*^ the binormal component gives rise to a logarithmic singularity and, to leading order, is responsible for the drift velocity of the vortex. It is therefore important to focus first on this contribution alone. From (6) we have:

7 $${v}_{b}({{\bf{x}}}_{o})={\bf{v}}({{\bf{x}}}_{o})\cdot \hat{{\bf{b}}}=\frac{\varGamma }{4\pi \rho }\,{\rm{l}}{\rm{n}}\frac{\rho }{\sigma }+C,$$

where *C* = [*Γ*/(4*πρ*)]*F* + **G** · $\hat{{\bf{b}}}$ is now function of the sole geometry and topology of ${{\mathscr{T}}}_{p,q}$. Thus, we have

8 $$C=C(\lambda ,w)={\bf{v}}({{\bf{x}}}_{o})\cdot \hat{{\bf{b}}}-\frac{\varGamma }{4\pi \rho }\,{\rm{l}}{\rm{n}}\frac{\rho }{\sigma }\,.$$

## Analytical De-Singularization of the Biot-Savart Integral

**Figure 2:**
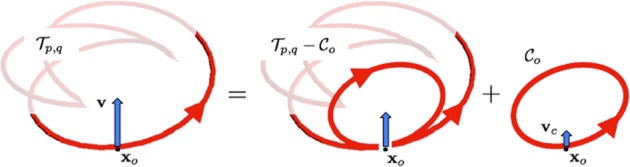
Application of Moore-Saffman’s de-singularization technique: the self-induced velocity **v** = **v**(**x**_*o*_) of ${{\mathscr{T}}}_{p,q}$ is given by the Biot-Savart integral over ${{\mathscr{T}}}_{p,q}-{{\mathscr{C}}}_{o}$ plus the velocity **v**_*c*_ of the osculating vortex ring ${{\mathscr{C}}}_{o}$ at **x**_*o*_.

In order to compute *C* = *C*(*λ*, *w*) from Eq. (8) we must first deal with the analytic logarithmic singularity that arises when **x**_*o*_ → x^*^. Since this singularity has no physical justification, but it is purely an artifact of the analytical expression (3), a de-singularization technique must be applied. For this we follow the prescription of Moore-Saffman^21^. This is based on the application of an asymptotic technique that matches the local shape and vorticity distribution of the given vortex with those of the osculating vortex ring of same local curvature *c* = *ρ*^−1^ and vorticity so to reproduce locally the correct dynamics. It relies on the observation that the streamline pattern induced by the local vortex geometry and core structure is, to leading order, the same as that obtained by replacing a strand of the original vortex with that of a vortex ring of same curvature and core structure. Indeed one can prove^28,29^ that by direct application of this method the error is at most of the order *O*(*δ*^2^), where *δ* = (*σ*/*R*)ln(8*R*/*σ*) and $\sigma /R\ll 1$. The singularity is thus removed by virtually subtracting and adding the contribution to the induced velocity at **x**_*o*_, replacing the original vortex with that of the osculating vortex ring ${{\mathscr{C}}}_{o}$ at **x**_*o*_ (see Fig. 2).

By exploiting the rotational symmetry of torus knots we consider the equatorial point **x**_*o*_ = (*R*(1 + *λ*), 0, 0); we have

9 $${\bf{v}}({{\bf{x}}}_{o})=\frac{\varGamma }{4\pi }[{\oint }_{{{\mathscr{T}}}_{p,q}}\frac{{\hat{{\bf{t}}}}_{k}\times ({{\bf{x}}}_{o}-{{\bf{x}}}_{k}^{\ast })}{|{{\bf{x}}}_{o}-{{\bf{x}}}_{k}^{\ast }{|}^{3}}\,{\rm{d}}{{\bf{x}}}_{k}^{\ast }-{\oint }_{{{\mathscr{C}}}_{o}}\frac{{\hat{{\bf{t}}}}_{c}\times ({{\bf{x}}}_{o}-{{\bf{x}}}_{c}^{\ast })}{|{{\bf{x}}}_{o}-{{\bf{x}}}_{c}^{\ast }{|}^{3}}{\rm{d}}{{\bf{x}}}_{c}^{\ast }]+{{\bf{v}}}_{c}({{\bf{x}}}_{o}),$$

where **x**_k_^*^ denotes a point on the knot and ${{\bf{x}}}_{{c}}^{\ast }$ a point on the osculating ring ${{\mathscr{C}}}_{o}$; **v**_*c*_(**x**_*o*_) is then given by Kelvin’s formula^30^ for uniform vorticity, i.e.

10 $${{\bf{v}}}_{c}({{\bf{x}}}_{o})=\frac{\varGamma }{4\pi \rho }\,[{\rm{l}}{\rm{n}}\frac{\rho }{\sigma }+\,{\rm{l}}{\rm{n}}\,8-\frac{1}{4}]\,\hat{{\bf{b}}}.$$

Let us calculate the parametric equations of ${{\mathscr{C}}}_{o}$. The Frenet frame at **x**_*o*_ is given by

11 $$\hat{{\bf{t}}}={A}^{-1}(0,1+\lambda ,\lambda w),\,\hat{{\bf{n}}}=(-1,0,0),\,\hat{{\bf{b}}}={A}^{-1}(0,-\,\lambda w,1+\lambda ),$$

where *A* = *A*(*λ*, *w*) = [(1 + *λ*)^2^ + *λ*^2^*w*^2^]^1/2^, and

12 $$\bar{\rho }=\frac{\rho }{R}=\frac{{(1+\lambda )}^{2}+{\lambda }^{2}{w}^{2}}{1+\lambda +\lambda {w}^{2}}.$$

Let *K* = *K*(*λ*, *w*) = 1 + *λ* + *λw*^2 ^; the vector position of points on the osculating circle ${{\mathscr{C}}}_{o}$ is given by **x**_c_^*^ = *O* + *ρ*($\hat{{\bf{t}}}$sin*ϑ* − $\hat{{\bf{n}}}$ cos*ϑ*) with $\vartheta \in [0,2\pi )$, i.e.

13 $${{\bf{x}}}_{c}^{\ast }=R{K}^{-1}(\lambda {w}^{2}+{A}^{2}\,\cos \,\theta ,A\mathrm{(1}+\lambda )\sin \,\theta ,A\lambda w\,\sin \,\theta ),$$

with centre at *O* = ${{\bf{x}}}_{{c}}^{\ast }$ + *ρ*$\hat{{\bf{n}}}$ = (*Rλw*^2^/*K*, 0, 0). By substituting the expressions above into (9), we have

14 $$\begin{array}{cc}{\bf{v}}({{\bf{x}}}_{o})= & \frac{\Gamma }{4\pi }\,[{\int }_{0}^{2\pi p}\frac{{\hat{{\bf{t}}}}_{k}(\alpha )\times ({{\bf{x}}}_{o}-{{\bf{x}}}_{k}^{\ast }(\alpha ))}{|{{\bf{x}}}_{o}-{{\bf{x}}}_{k}^{\ast }(\alpha {)|}^{3}}|\frac{{\rm{d}}{{\bf{x}}}_{k}^{\ast }(\alpha )}{{\rm{d}}\alpha }|{\rm{d}}\alpha \\ & -{\int }_{0}^{2\pi }\frac{{\hat{{\bf{t}}}}_{c}(\vartheta )\times ({{\bf{x}}}_{o}-{{\bf{x}}}_{c}^{\ast }(\vartheta ))}{|{{\bf{x}}}_{o}-{{\bf{x}}}_{c}^{\ast }(\vartheta {)|}^{3}}|\frac{{\rm{d}}{{\bf{x}}}_{c}^{\ast }(\vartheta )}{{\rm{d}}\vartheta }|{\rm{d}}\vartheta ]+{{\bf{v}}}_{c}({{\bf{x}}}_{o}).\end{array}$$

## Winding Number Effects on the Self-Induced Velocity

We can now determine the influence of the winding number on the self-induced motion of the vortex. Using (10) and by renaming *ϑ*  to  *α*, the binormal component is given by

15 $${v}_{b}({{\bf{x}}}_{o})=\frac{\varGamma }{4\pi }\,[{\int }_{0}^{2\pi p}{\mathscr{B}}{{\mathscr{S}}}_{k}\,{\rm{d}}\alpha -{\int }_{0}^{2\pi }{\mathscr{B}}{{\mathscr{S}}}_{c}\,{\rm{d}}\alpha ]+{v}_{c}({{\bf{x}}}_{o}),$$

where

16 $$ {\mathcal B} {{\mathscr{S}}}_{k}=\frac{{\hat{{\bf{t}}}}_{k}(\alpha )\times ({{\bf{x}}}_{o}-{{\bf{x}}}_{k}^{\ast }(\alpha ))\cdot \hat{{\bf{b}}}}{|{{\bf{x}}}_{o}-{{\bf{x}}}_{k}^{\ast }(\alpha {)|}^{3}}|\frac{{\rm{d}}{{\bf{x}}}_{k}^{\ast }(\alpha )}{{\rm{d}}\alpha }|,$$

and

17 $${\mathscr{B}}{{\mathscr{S}}}_{c}=\frac{{\hat{{\bf{t}}}}_{c}(\alpha )\times ({{\bf{x}}}_{o}-{{\bf{x}}}_{c}^{\ast }(\alpha ))\cdot \hat{{\bf{b}}}}{|{{\bf{x}}}_{o}-{{\bf{x}}}_{c}^{\ast }(\alpha {)|}^{3}}|\frac{{\rm{d}}{{\bf{x}}}_{c}^{\ast }}{{\rm{d}}\alpha }|,$$

refer respectively to the points on ${{\mathscr{T}}}_{p,q}$ and ${{\mathscr{C}}}_{o}$.

### Lemma 1.

*The functions* ${\mathcal B} {{\mathscr{S}}}_{k}= {\mathcal B} {{\mathscr{S}}}_{k}(\alpha ;\lambda ,w)$ *and* ${\mathcal B} {{\mathscr{S}}}_{c}= {\mathcal B} {{\mathscr{S}}}_{c}(\alpha ;\lambda ,w)$ *have*, *to leading order near the point* **x** = **x**_*o*_, *the same functional dependence on α*, *given by*

18 $$ {\mathcal B} {\mathscr{S}}=\frac{1}{2\rho \alpha }+\mathop{\sum }\limits_{i=0}^{+\infty }\,{b}_{2i+1}{\alpha }^{2i+1},$$

*where b*_2*i*+1_ *are functions of the local geometry of the curve*.

*Proof*. Let’s take Taylor’s expansion of the numerator *N* and denominator *D* of the functions ${\mathcal B} {{\mathscr{S}}}_{k}$ and ${\mathcal B} {{\mathscr{S}}}_{c}$ near *α* = 0. Consider the formal expansion of *N*/*D* given by

19 $$\frac{N}{D}=\mathop{\sum }\limits_{i=-\infty }^{+\infty }\,{b}_{i}{\alpha }^{i}\,,$$

in the unknowns *b*_*i*_ (to be determined). Let’s equate the series expansion of the numerator *N* to (*N*/*D*)*D* obtained by multiplying (19) with the expansion of the denominator *D* of the corresponding function. By matching coefficients of same power of *α* we have that *b*_*i*_ = 0 for any *i* < −1 and *i* ≥ 2*n*, *n* = 0, 1, 2, …. Moreover *b*_−1_ = (2*ρ*)^−1^, hence to leading order in *α* the two integrands have same singurality and functional behavior.

Now, note that ${\mathcal B} {{\mathscr{S}}}_{k}$ and ${\mathcal B} {{\mathscr{S}}}_{c}$ are even functions of *α*, so that (15) is reduced to

20 $$\begin{array}{rcl}{v}_{b}({{\bf{x}}}_{o}) & = & \frac{\Gamma }{2\pi }[{\int }_{0}^{\pi p} {\mathcal B} {{\mathscr{S}}}_{k}\,{\rm{d}}\alpha -{\int }_{0}^{\pi } {\mathcal B} {{\mathscr{S}}}_{c}\,{\rm{d}}\alpha ]+{v}_{c}({{\bf{x}}}_{o})\\ & = & \frac{\Gamma }{4\pi }({I}_{1}+{I}_{2})+{v}_{c}({{\bf{x}}}_{o}\mathrm{).}\end{array}$$

where

21 $${I}_{1}=2{\int }_{0}^{\pi }( {\mathcal B} {{\mathscr{S}}}_{k}- {\mathcal B} {{\mathscr{S}}}_{c})\,{\rm{d}}\alpha ,\,\,{I}_{2}=2{\int }_{\pi }^{\pi p} {\mathcal B} {{\mathscr{S}}}_{k}{\rm{d}}\alpha \mathrm{.}$$

### Binormal component of the self-induced velocity

From (20) and (10) we have

22 $${\bar{v}}_{b}({{\bf{x}}}_{o})=\frac{{v}_{b}({{\bf{x}}}_{o})}{\varGamma /4\pi R}=R({I}_{1}+{I}_{2})+\frac{R}{\rho }\,[{\rm{l}}{\rm{n}}\frac{\rho }{\sigma }+\,{\rm{l}}{\rm{n}}\,8-\frac{1}{4}],$$

and by comparing the above with (8), we obtain

23 $${\bar{v}}_{b}({{\bf{x}}}_{o})=\frac{R}{\rho }\,{\rm{l}}{\rm{n}}\frac{\rho }{\sigma }+\bar{C},$$

with

24 $$\bar{C}=\bar{C}(\lambda ,w)=R({I}_{1}+{I}_{2})+\frac{R}{\rho }(\mathrm{ln}\,8-\frac{1}{4}).$$

**Figure 3:**
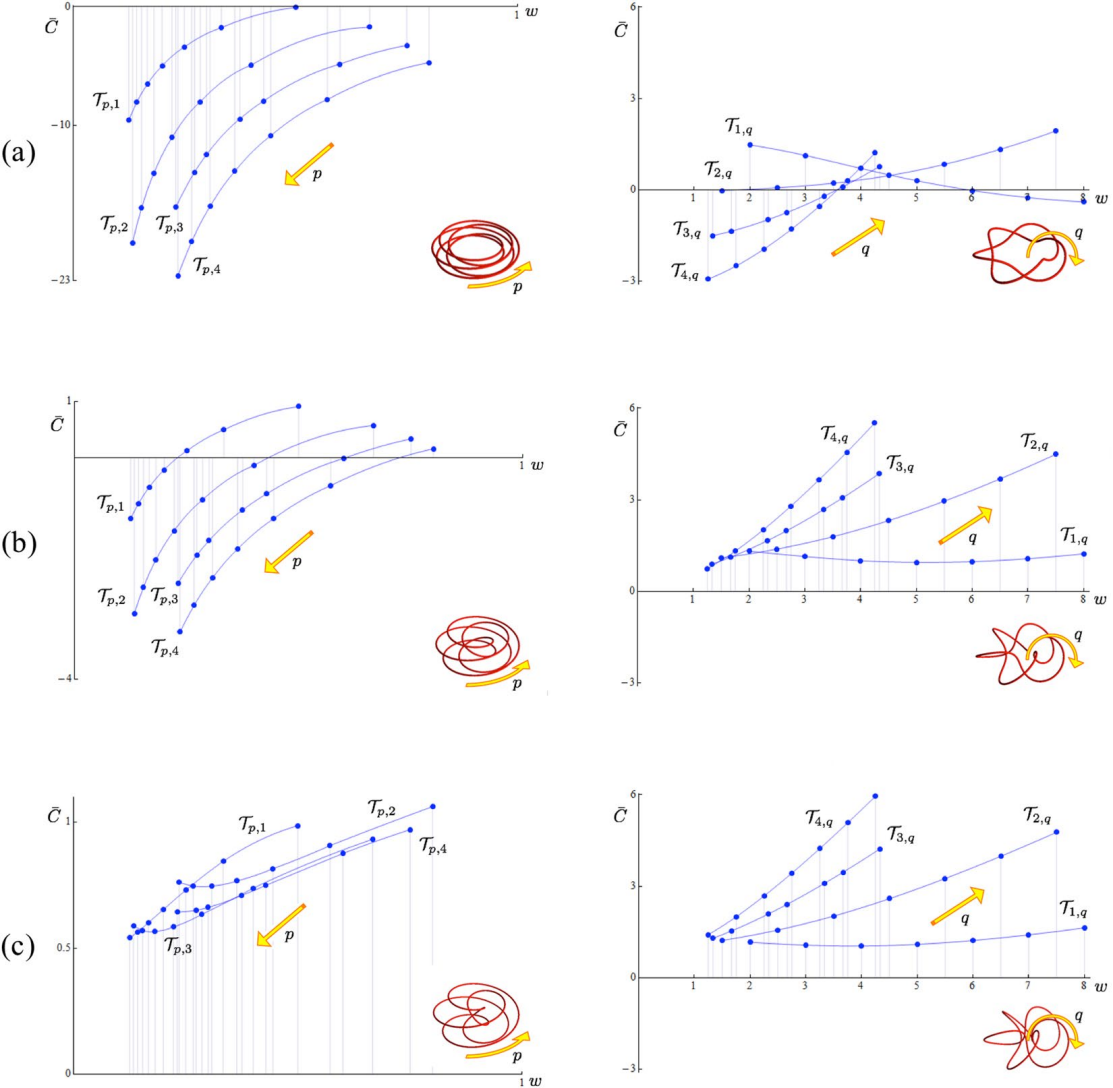
Contributions to the binormal component of the drift velocity at ${{\bf{x}}}_{{o}}$,$\bar{C}$ = $\bar{C}$(*λ*, *w*) from Eq. (24), plotted against *w*: (**a**) *λ* = 0.25, (**b**) *λ* = 0.5, (**c**) *λ* = 0.75 and *R* = 1. Toroidal types (*p* > *q*) are on the left, poloidal types (*p* < *q*) on the right. ${{\mathscr{T}}}_{p\mathrm{,1}}$ and ${{\mathscr{T}}}_{\mathrm{1,}q}$ (*p*, *q* = 2, 3, 4, 5, 6, 7, 8); ${{\mathscr{T}}}_{p\mathrm{,2}}$ and ${{\mathscr{T}}}_{\mathrm{2,}q}$ (*p*, *q* = 3, 5, 7, 9, 11, 13, 15); ${{\mathscr{T}}}_{p\mathrm{,3}}$ and ${{\mathscr{T}}}_{\mathrm{3,}q}$ (*p*, *q* = 4, 5, 7, 8, 10, 11, 13); ${{\mathscr{T}}}_{p\mathrm{,4}}$ and ${{\mathscr{T}}}_{\mathrm{4,}q}$ (*p*, *q* = 5, 7, 9, 11, 13, 15, 17). Interpolation is for visualization purposes only. The dominant geometry is shown in inset for visualization purposes only.

Contributions to the binormal component $\bar{C}$ = $\bar{C}$(*λ*, *w*) of 42 torus knots and 14 unknots have been examined for various aspect ratios and increasing values of *w*. The plots are shown in Fig. 3 for (a) *λ* = 0.25, (b) *λ* = 0.5, (c) *λ* = 0.75 and *R* = 1. Plots on the left show the contributions from toroidal knots and unknots (*p* > *q*, *w* < 1); plots on the right show the contributions from poloidal knots and unknots (*q* > *p*, *w* > 1). As we see $\bar{C}$ tends to increase with *λ*, regardless of the knot type; for a given aspect ratio, $\bar{C}$ generically increases with decreasing number of toroidal coils and increasing number of poloidal coils. From Eq. (23) we notice that this represents only part of the total contribution to the binormal component, since winding number effects are also present in the first term of Eq. (23) (see the influence of *w* on local curvature in^23^).

### Tangential and normal components of the self-induced velocity

As mentioned before the tangential and normal contributions to the velocity are not singular. Hence, replacing $\hat{{\bf{b}}}$ into (16) first by  $\hat{{\bf{t}}}$ and then by $\hat{{\bf{n}}}$ we have ${\mathcal B} {{\mathscr{S}}}_{k}^{t}$ and ${\mathcal B} {{\mathscr{S}}}_{k}^{n}$, that after integration over [0, 2*πp*] give respectively *v*_*t*_(**x**_*o*_) and *v*_*n*_(**x**_*o*_). The contribution ${\bar{C}}_{t}$ to the tangential component of the velocity at **x**_*o*_ is given by considering the tangential component of Eq. (6); combining this latter with Eq. (15), where we replace ${\mathcal B} {{\mathscr{S}}}_{k}$ with ${\mathcal B} {{\mathscr{S}}}_{k}^{t}$, we have

25 $${\bar{C}}_{t}={\bar{C}}_{t}(\lambda ,w)={\overline{v}}_{t}({{\bf{x}}}_{o})=\frac{{v}_{t}({{\bf{x}}}_{o})}{\varGamma /4\pi R}=R{I}_{{\rm{1}}t},$$

where now

26 $${I}_{1t}=2{\int }_{0}^{\pi p} {\mathcal B} {{\mathscr{S}}}_{k}^{t}\,{\rm{d}}\alpha =2{\int }_{0}^{\pi p}\frac{{\hat{{\bf{t}}}}_{k}(\alpha )\times ({{\bf{x}}}_{o}-{{\bf{x}}}_{k}^{\ast }(\alpha ))\cdot \hat{{\bf{t}}}}{|{{\bf{x}}}_{o}-{{\bf{x}}}_{k}^{\ast }(\alpha {)|}^{3}}|\frac{{\rm{d}}{{\bf{x}}}_{k}^{\ast }(\alpha )}{{\rm{d}}\alpha }|{\rm{d}}\alpha \mathrm{.}$$

**Figure 4:**
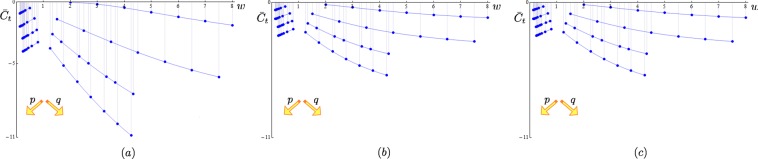
Contributions to the tangential component of the drift velocity at ${{\bf{x}}}_{{o}}$, ${\bar{C}}_{t}$ = ${\bar{C}}_{t}$(*λ*, *w*) from Eq. (25), plotted against *w*: (**a**) *λ* = 0.25, (**b**) *λ* = 0.5, (**c**) *λ* = 0.75 and *R* = 1. Toroidal types (*p* > *q*) are on the left-hand side of each diagram, poloidal types (*p* < *q*) on the right. ${{\mathscr{T}}}_{p\mathrm{,1}}$ and ${{\mathscr{T}}}_{\mathrm{1,}q}$ (*p*, *q* = 2, 3, 4, 5, 6, 7, 8); ${{\mathscr{T}}}_{p\mathrm{,2}}$ and ${{\mathscr{T}}}_{\mathrm{2,}q}$ (*p*, *q* = 3, 5, 7, 9, 11, 13, 15); ${{\mathscr{T}}}_{p\mathrm{,3}}$ and ${{\mathscr{T}}}_{\mathrm{3,}q}$ (*p*, *q* = 4, 5, 7, 8, 10, 11, 13); ${{\mathscr{T}}}_{p\mathrm{,4}}$ and ${{\mathscr{T}}}_{\mathrm{4,}q}$ (*p*, *q* = 5, 7, 9, 11, 13, 15, 17). Interpolation is for visualization purposes only.

Plots of ${\bar{C}}_{t}$ = ${\bar{C}}_{t}$(*λ*, *w*) against  different values of aspect ratio and winding number are shown in Fig. 4. As we see |${\bar{C}}_{t}$| generically increases with increasing *p* and *q*, leading to an increase of orbital motion of the knot around the torus central and circular axes. By direct inspection we can also see that ${\mathcal B} {{\mathscr{S}}}_{k}^{n}$ is anti-symmetric with respect to the interval of integration, i.e. ${\mathcal B} {{\mathscr{S}}}_{k}^{n}(\pi p-\alpha )=-\, {\mathcal B} {{\mathscr{S}}}_{k}^{n}(\pi p+\alpha )$; hence *v*_*n*_(**x**_*o*_) = 0.

### Global contributions to the velocity

The vortex translates in the fluid in the direction of the torus central axis with a speed *U*, and it is also subject to a combined action of a rigid rotation *Θ* around the *z*–axis and an orbital rigid rotation *Ω* in the meridian direction around the torus circular axis *R* = 1. *Θ* and *Ω* do not contribute to displace the vortex center of mass, and the direction of these contributions is reversed if we reverse the orientation of vorticity, i.e. if we change *α* → −*α* in (1). Since torus knots are chiral knots^23^, by changing z^*^ → −z^*^ in (1) left-handed knots are transformed into right-handed knots. Chirality implies a reversal of *Ω*, but it has no effect on the directions of *Θ* and *U*.

#### Translation speed

Let us consider the translation speed *v*_*z*_(**x**_*o*_) = **v**(**x**_*o*_) · ${\hat{{\bf{e}}}}_{z}$ at **x**_*o*_ in the *z* direction. From (14) we have

27 $$\begin{array}{ccc}{v}_{z} & = & \frac{\varGamma }{4\pi }\,[{\int }_{0}^{2\pi p}\frac{{\hat{{\bf{t}}}}_{k}(\alpha )\times ({{\bf{x}}}_{o}-{{\bf{x}}}_{k}^{\ast }(\alpha ))\cdot {\hat{{\bf{e}}}}_{z}}{|{{\bf{x}}}_{o}-{{\bf{x}}}_{k}^{\ast }(\alpha {)|}^{3}}|\frac{{\rm{d}}{{\bf{x}}}_{k}^{\ast }(\alpha )}{{\rm{d}}\alpha }|{\rm{d}}\alpha \\ & & -{\int }_{0}^{2\pi }\frac{{\hat{{\bf{t}}}}_{c}(\vartheta )\times ({{\bf{x}}}_{o}-{{\bf{x}}}_{c}^{\ast }(\vartheta ))\cdot {\hat{{\bf{e}}}}_{z}}{|{{\bf{x}}}_{o}-{{\bf{x}}}_{c}^{\ast }(\vartheta {)|}^{3}}|\frac{{\rm{d}}{{\bf{x}}}_{c}^{\ast }(\vartheta )}{{\rm{d}}\vartheta }|{\rm{d}}\vartheta ]+{{\bf{v}}}_{c}({{\bf{x}}}_{o})\cdot {\hat{{\bf{e}}}}_{z}.\end{array}$$

Taking advantage of the symmetry properties of the integrands, we have

28 $${\bar{v}}_{z}=\frac{{v}_{z}}{\varGamma /4\pi R}=\frac{R}{\rho }\,{\rm{l}}{\rm{n}}\frac{\rho }{\sigma }\,\hat{{\bf{b}}}\cdot {\hat{{\bf{e}}}}_{z}+{\bar{C}}_{z},$$

with

29 $${\bar{C}}_{z}=R({I}_{1z}+{I}_{2z})+\frac{R}{\rho }\,({\rm{l}}{\rm{n}}\,8-\frac{1}{4})\,\hat{{\bf{b}}}\cdot {\hat{{\bf{e}}}}_{z},$$

where

30 $$\begin{array}{rcl}{I}_{1z} & = & 2{\int }_{0}^{\pi }[\frac{{\hat{{\bf{t}}}}_{k}(\alpha )\times ({{\bf{x}}}_{o}-{{\bf{x}}}_{k}^{\ast }(\alpha ))}{|{{\bf{x}}}_{o}-{{\bf{x}}}_{k}^{\ast }(\alpha {)|}^{3}}|\frac{{\rm{d}}{{\bf{x}}}_{k}^{\ast }(\alpha )}{{\rm{d}}\alpha }|\\ & & -\frac{{\hat{{\bf{t}}}}_{c}(\alpha )\times ({{\bf{x}}}_{o}-{{\bf{x}}}_{c}^{\ast }(\alpha ))}{|{{\bf{x}}}_{o}-{{\bf{x}}}_{c}^{\ast }(\alpha {)|}^{3}}|\frac{{\rm{d}}{{\bf{x}}}_{c}^{\ast }(\alpha )}{{\rm{d}}\alpha }|]\cdot {\hat{{\bf{e}}}}_{z}\,{\rm{d}}\alpha ,\end{array}$$

and

31 $${I}_{2z}=2{\int }_{\pi }^{\pi p}\frac{{\hat{{\bf{t}}}}_{k}(\alpha )\times ({{\bf{x}}}_{o}-{{\bf{x}}}_{k}^{\ast }(\alpha ))\cdot {\hat{{\bf{e}}}}_{z}}{|{{\bf{x}}}_{o}-{{\bf{x}}}_{k}^{\ast }(\alpha {)|}^{3}}|\frac{{\rm{d}}{{\bf{x}}}_{k}^{\ast }(\alpha )}{{\rm{d}}\alpha }|\,{\rm{d}}\alpha \mathrm{.}$$

**Figure 5:**
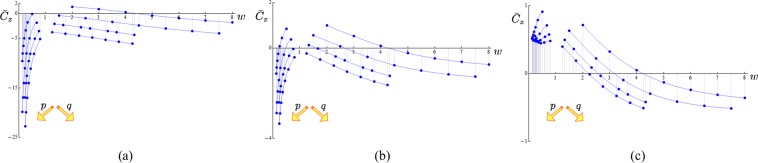
Finite term contribution to the translation speed at the point ${{\bf{x}}}_{{o}}$, ${\bar{C}}_{z}$ = ${\bar{C}}_{z}$(*λ*, *w*) from Eq. (29), plotted against *w*: (**a**) *λ* = 0.25, (**b**) *λ* = 0.5, (**c**) *λ* = 0.75 and *R* = 1. Toroidal types (*p* > *q*) are on the left-hand-side of each diagram, poloidal types (*p* < *q*) on the right. ${{\mathscr{T}}}_{p\mathrm{,1}}$ and ${{\mathscr{T}}}_{\mathrm{1,}q}$ (*p*, *q* = 2, 3, 4, 5, 6, 7, 8); ${{\mathscr{T}}}_{p\mathrm{,2}}$ and ${{\mathscr{T}}}_{\mathrm{2,}q}$ (*p*, *q* = 3, 5, 7, 9, 11, 13, 15); ${{\mathscr{T}}}_{p\mathrm{,3}}$ and ${{\mathscr{T}}}_{\mathrm{3,}q}$ (*p*, *q* = 4, 5, 7, 8, 10, 11, 13); ${{\mathscr{T}}}_{p\mathrm{,4}}$ and ${{\mathscr{T}}}_{\mathrm{4,}q}$ (*p*, *q* = 5, 7, 9, 11, 13, 15, 17). Interpolation is for visualization purposes only.

Plots of ${\bar{C}}_{z}$ = ${\bar{C}}_{z}$(*λ*, *w*) versus *w* are shown in Fig. 5. As mentioned, two simultaneous motions take place: one along the meridian direction associated with the uniform orbital motion of the knot strands around the torus circular axis *R* = 1; the other along the *z*-axis responsible for the translation speed *U* of the vortex in the fluid. As we see from the plots ${\bar{C}}_{z}$ may change sign for particular values of winding number and aspect ratio. It’s easy to determine whether this decrease may lead to a local standstill, or even a reverse motion; from (28), we have

32 $${\bar{v}}_{z}({{\bf{x}}}_{o})=\frac{R}{\rho }\,{\rm{l}}{\rm{n}}\frac{\rho }{\sigma }\,\hat{{\bf{b}}}\cdot {\hat{{\bf{e}}}}_{z}+{\bar{C}}_{z}\le 0\,\,\Longleftrightarrow \,\,{\bar{C}}_{z}\le \frac{R}{\rho }\,{\rm{l}}{\rm{n}}\frac{\sigma }{\rho }\,\hat{{\bf{b}}}\cdot {\hat{{\bf{e}}}}_{z}.$$

This provides an analytical condition for the reversal of the translation velocity of vortex knots and unknots; in particular, as we see from the plots of the poloidal coils of Fig. 5 (with either *p* = 1 or *q* = 1), we have proof of the anomalous translation of perturbed vortex rings  observed numerically in superfluids^31^; the results of Fig. 5 for *p* = 1 and increasing *q* show that indeed the reversed speed increases with aspect ratio (that corresponds to wave amplitude in Fig. 1(d) of^31^), and for a given aspect ratio it increases with the number *q* of poloidal coils (that corresponds to the number of waves in Fig. 2 of^31^). Our results provide also mathematical  grounds to the observed reversed motion of *viscous* vortex rings in presence of intense swirl^32^. Since swirling flow is generated by a bundle of poloidal vortex lines confined to a ring, high swirl corresponds to high number of poloidal coils (with *p* = 1), leading to the condition (32) discussed above.

The translation speed *U* can be computed explicitly for particular knot configurations. For the family ${{\mathscr{T}}}_{2,q}$ (*q* = 3, 5, …, 15), let us consider the algebraic mean of the induced velocities at various points in the meridian plane^33^. The normalized speed $\bar{U}$ is thus defined by

33 $$\bar{U}=\frac{[{\bar{v}}_{z}{]}_{\alpha =0}+[{\bar{v}}_{z}{]}_{\alpha =2\pi }}{2}.$$

**Figure 6:**
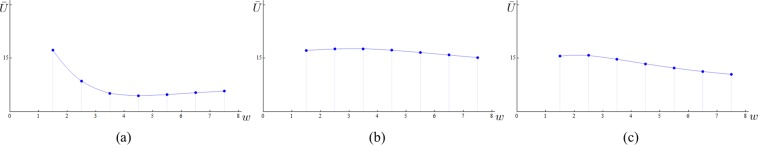
Normalized translation speed $\bar{U}$ = $\bar{U}$(*w*) from Eq. (33) plotted against *w* for torus knots ${{\mathscr{T}}}_{2,q}$ (*q* = 3, 5, …, 15) with *σ* = 10^−8^, *R* = 1; (**a**) *λ* = 0.25, (**b**) *λ* = 0.50, (**c**) *λ* = 0.75.

**Figure 7:**
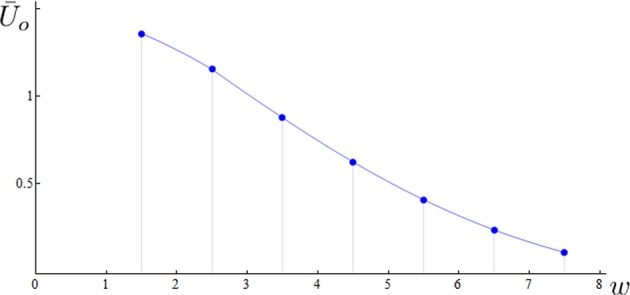
Normalized translation speed ${\bar{U}}_{o}$ = $\bar{U}$/*lnδ* plotted against *w* for torus knots ${{\mathscr{T}}}_{2,q}$ (*q* = 3, 5, …, 15) with *σ* = 10^−8^, *R* = 1, *ln δ* = 18.61 and *λ* = 0.1.

Numerical integration at the innermost  points presents some difficulty due the appearance of numerical instabilities associated with the concentration of the streamlines in the toroidal region^33^. Plots of $\bar{U}$ = $\bar{U}$(*w*) are shown in Fig. 6 for extremely thin vortex cores. As was noted before^8,20^ the vortex speed increases with the number of toroidal coils *p*, but here we notice also secondary effects due to *q*. The order of magnitude of $\bar{U}$ is only slightly influenced by *q* when *λ* ≥ 0.5, but it changes considerably when *λ* ≤ 0.3. This behavior went  unnoticed in^8^. Partial comparison with results obtained in superfluids  can be made by taking ${\bar{U}}_{o}$ =$\bar{U}$/*lnδ*, with *λ* = 0.1, *σ* = 10^−8^ and ln *δ*= 18.61 (typical values for superfluid vortices). Computations of ${\bar{U}}_{o}$ = ${\bar{U}}_{o}$(*w*) for ${{\mathscr{T}}}_{2,q}$ (*q* = 3, 5, 7, 9) are shown in Fig. 7. Direct comparison with data obtained by^20^ (Fig. 5, ${{\mathscr{T}}}_{2,q}$ for *q* = 3, 5, 7, 9) is limited by the assumption of superfluid hollow vortex core. The general trend of a decrease in propagation speed as function of *w* is confirmed, but in the present case of uniform vorticity core distribution we observe a much more drastic decrease of ${\bar{U}}_{o}$ for increasing *w*. This result is consistent with higher values of rotational energy that at high *q* induce a much stronger toroidal jet inside *Π*, with a consequential further reduction of the overall speed of the vortex.

## Concluding Remarks

By using the Moore-Saffman de-singularization technique we have studied the self-induced motion of vortex torus knots ${{\mathscr{T}}}_{p,q}$ under the full Biot-Savart law. This has been done by analyzing 56 different knots and unknots up to 51 crossings, determining the precise relationships between winding number and velocity contributions. We can state that the influence of winding number has leading order effects on vortex motion,  comparable to curvature effects. The effects of *w* = *q*/*p* are related to the relative number of toroidal and poloidal coils. For complex knots the number of toroidal coils *p* is in general of primary importance. For given *p* and *q* knots travel faster than unknots, and if *p* > *q* ${{\mathscr{T}}}_{p,q}$ knots travel faster than ${{\mathscr{T}}}_{q,p}$ knots. In particular we prescribe the condition for a reversal of the translation speed, providing theoretical grounds for the numerically observed reversal of vortex rings subject to superimposed large-amplitude perturbations^31^ or swirl^32^. We can also establish an increased  influence of poloidal coils on the propagation of thin-cored vortex knots that went unnoticed in previous numerical works^8^ or that resulted in a much milder effects for superfluid hollow vortex cores^20^.

These new findings help to interpret the evolution of three-dimensional, localized bundle of vortex lines^34,35^ in complex networks of vortical flows, where the coiling of field lines has a primary effect on the motion. Indeed, whereas the pitch of an isolated helix was found to have only a secondary effect on vortex motion^22^, the helical winding of vortex lines, due to the combined effects of poloidal and toroidal coils through *w* (see Eq. (12) above), can actually be of primary importance. Since in reconnection processes twist effects are important for helicity considerations and energy transfers across scales^36,37^, these results are important to understand the role of three-dimensional features in the evolution and coherence of localized vortical flows.
